# The plastid and mitochondrial genomes of *Eucalyptus grandis*

**DOI:** 10.1186/s12864-019-5444-4

**Published:** 2019-02-13

**Authors:** Desre Pinard, Alexander A. Myburg, Eshchar Mizrachi

**Affiliations:** 1 Department of Biochemistry, Genetics and Microbiology, Forestry and Agricultural Biotechnology Institute (FABI), University of Pretoria, Private Bag X20, Pretoria, 0028 South Africa; 2 Genomics Research Institute (GRI), University of Pretoria, Private Bag X20, Pretoria, 0028 South Africa

**Keywords:** *Eucalyptus grandis*, Organelle genome, Mitochondria, Chloroplast, Plastid

## Abstract

**Background:**

Land plant organellar genomes have significant impact on metabolism and adaptation, and as such, accurate assembly and annotation of plant organellar genomes is an important tool in understanding the evolutionary history and interactions between these genomes. Intracellular DNA transfer is ongoing between the nuclear and organellar genomes, and can lead to significant genomic variation between, and within, species that impacts downstream analysis of genomes and transcriptomes.

**Results:**

In order to facilitate further studies of cytonuclear interactions in *Eucalyptus*, we report an updated annotation of the *E. grandis* plastid genome, and the second sequenced and annotated mitochondrial genome of the Myrtales, that of *E. grandis*. The 478,813 bp mitochondrial genome shows the conserved protein coding regions and gene order rearrangements typical of land plants. There have been widespread insertions of organellar DNA into the *E. grandis* nuclear genome, which span 141 annotated nuclear genes. Further, we identify predicted editing sites to allow for the discrimination of RNA-sequencing reads between nuclear and organellar gene copies, finding that nuclear copies of organellar genes are not expressed in *E. grandis*.

**Conclusions:**

The implications of organellar DNA transfer to the nucleus are often ignored, despite the insight they can give into the ongoing evolution of plant genomes, and the problems they can cause in many applications of genomics. Future comparisons of the transcription and regulation of organellar genes between *Eucalyptus* genotypes may provide insight to the cytonuclear interactions that impact economically important traits in this widely grown lignocellulosic crop species.

**Electronic supplementary material:**

The online version of this article (10.1186/s12864-019-5444-4) contains supplementary material, which is available to authorized users.

## Background

Plastid and mitochondrial genomes are well studied aspects of land plant genomics, with 2484 plastid (1987 “chloroplast”, 506 “plastid”), and 167 mitochondrial genomes on NCBI for land plants as of June 2018, compared to the 141 nuclear genomes completed at the chromosome level [1]. A consequence of the endosymbiotic integration of plastids and mitochondria into plant cells is that the coding potential of their genomes is severely diminished compared to their ancestral genomes [2, 3]. The majority of organellar proteomes are encoded by the nuclear genome of plants, with ±97% of plastid, and ± 99% of mitochondrial targeted proteins encoded by the nucleus [4]. Retained protein-coding organellar genes are essential to the metabolic functions of plastids and mitochondria, and variation in organellar genomes impact fitness and metabolism in angiosperms [5–9].

In plants, intracellular DNA transfer results in nuclear plastidial DNAs (NUPTs) and nuclear mitochondrial DNAs (NUMTs), that are still present in the organellar genomes [4]. Phylogenetic analysis of *Arabidopsis* and rice organellar DNA insertions show that large, primary insertions of organellar DNA into the nuclear genomes of plants occur, and these insertions decay over time [10]. The rate and distribution of organellar inserts into the nuclear genome vary between plant species, as do the location and proximity to transposable elements, which rearrange and expand inserted regions [10]. These recent inter-genomic DNA transfers between the nuclear and organellar genomes can result in multiple copies of organellar genes in the nuclear genome, presenting interesting avenues of research into the evolutionary history of plants and the process of endosymbiosis, as ongoing gene transfer may lead to the loss of the organellar encoded copy [11].

Key requirements to understanding the impact of organellar genome variation and transcript expression are high-quality annotated genomes, and a catalogue of intracellular genome transfers in order to distinguish between RNA originating from the organellar and nuclear genomes. Since it was sequenced in 2014, the *Eucalyptus* genome has become an important, and highly utilized genome for a variety of biological, ecological, and biotechnological studies [12]. Here, we update the assembly and annotation of the *E. grandis* plastid genome (adding 14 genes) and assemble and annotate the mitochondrial genome of *E. grandis*. We identified recent organellar genome transfers, and potential editing sites that can be used to distinguish transcripts originating from the organellar and nuclear genomes.

## Results

### Genome structure and gene content of the *E. grandis* mitochondrial genome

**Fig. 1:**
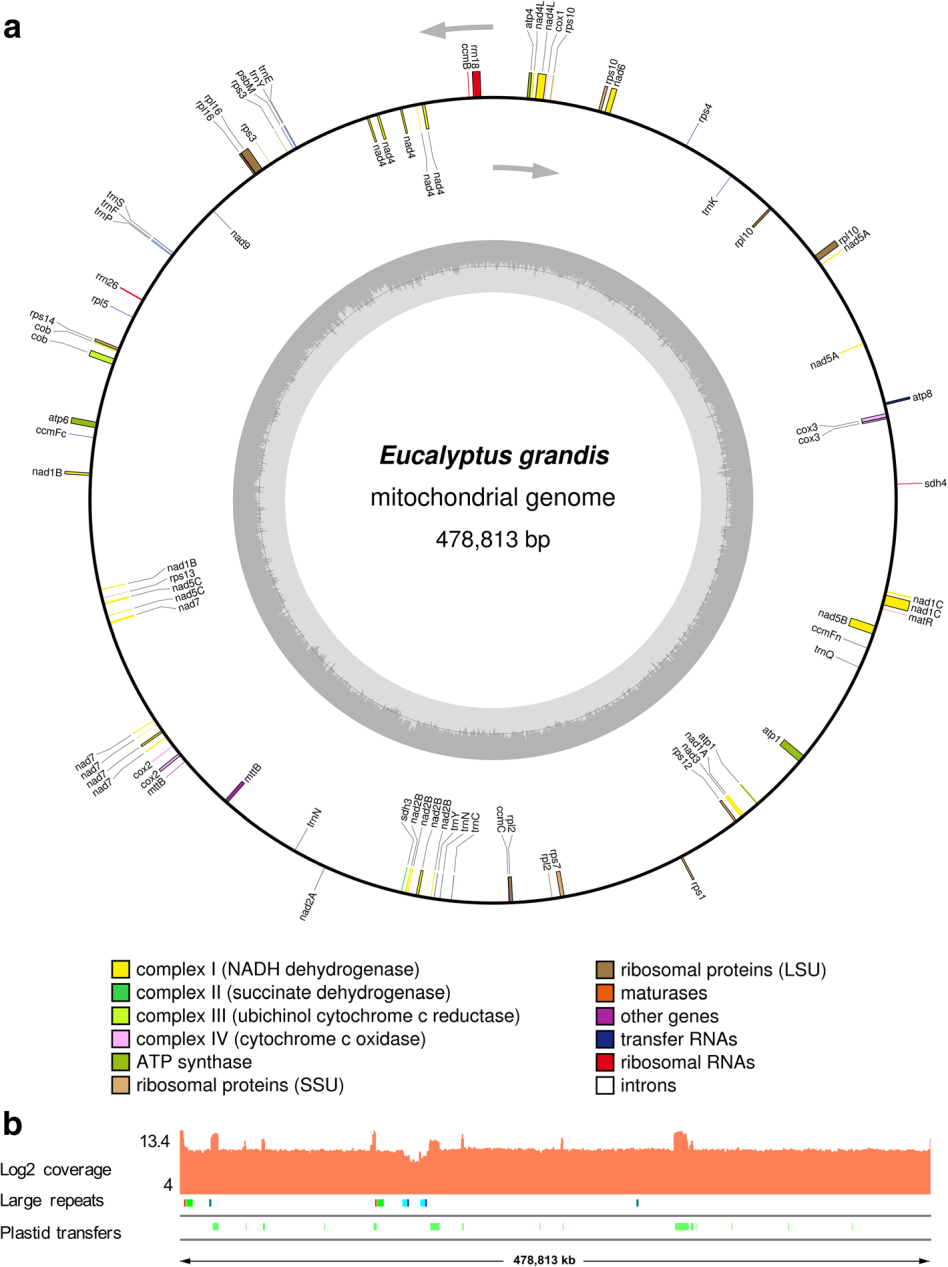
Mitochondrial genome of *E. grandis*. **a**. Genomic features are shown facing outward (positive strand) and inward (negative strand) of the *E. grandis* mitochondrial genome represented as a circular molecule. The colour key shows the functional class of the mitochondrial genes, and introns are shown in white. The GC content is represented in the innermost circle. The figure was generated in OGDraw [86]. **b**. Genome coverage of *E. grandis* WGS reads in log2 scale (Log coverage) across the mitochondrial genome. WGS reads were mapped with Bowtie 2 [77] and visualized in IGV [78]. The second track (Large repeats) shows mitochondrial repeat regions > 1000 bp in length, with pairs in matching colours, all repeat pairs are direct repeats, with the exception of the repeat pair shown in teal. The third track (Plastid transfers) shows plastid to mitochondrial DNA transfers longer than 100 bp, with e-value > 1 × 10^− 5^ in green

We used mitochondrial genome scaffolds from the Joint Genome Institute assembly of the *E. grandis* nuclear genome to perform a reference-based assembly of the mitochondrial genome from Illumina whole genome sequencing (WGS) data. The assembled mitochondrial genome is a single scaffold of 478,813 bp, with average GC content of 44.8% (Fig. 1a and b, Table 1). The average coverage of the WGS reads across the mitochondrial genome is ~ 700, with regions of ten times the average coverage representing overlaps between the plastid and mitochondrial genomes (Fig. 1b) Repeat elements make up 2.47% of the *E. grandis* mitochondrial genome, consisting mainly of simple and low complexity repeats (Table 1, Additional file 1: Table S1). We identified 19 direct repeat regions larger than 100 bp in the *E. grandis* mitochondrial genome, the largest of which is 4210 bp long (Fig. 1b, Additional file 2). Additionally, we identified 11 inverted repeat regions longer than 100 bp in the *E. grandis* mitochondrial genome, the largest of which is 1352 bp (Fig. 1b, Additional file 2). Due to the fact that we could not assemble a circular mitochondrial genome for *E. grandis* from whole genome sequencing data, we considered that the genome may indeed be present as a linear molecule, or as sub-genomic molecules that arise via recombination of the repeat regions [13]. We did not find evidence of sub-genomic molecules from the depth of coverage across the mitochondrial genome assembly (Fig. 1b). We used SVDetect to determine if any structural variations exist by filtering the alignment file based on the distance and orientation of aligned reads, along with removing any reads whose mate mapped to the nuclear or plastid genomes [14]. The SVDetect defined breakpoints were cross-referenced with the large repeat regions, and the results suggest that most repeat regions are not mediating mitochondrial sub-genomic molecules, as 8 breakpoints are within 250 bp of a repeat region and are found to predominantly be supported by less than 100 read pairs, with three being supported by 309, 219, and 163 read pairs (Additional file 3). Of these, direct repeat 13 shows evidence of repeat mediated structural variation, supported by 219 read pairs (Additional file 4: Figure S1). Any other alternate conformations present in the *E. grandis* mitochondrial genome could not be identified using this data and could be further assessed using long-read sequencing in the future.

**Table 1:** *E. grandis* organellar genome characteristics

Metric	Mitochondria	Plastid
Genome size (bp)	478,813	160,109
GC content	44.80%	36.90%
WGS coverage	~700x	~3456x
WGS insert size	475 bp	475 bp
WGS read length	100 bp	100 bp
Total length of homologous regions in the nuclear genome	1,256,558 bp	751,886 bp
% of inter-organellar DNA transfers	6% (28,123 bp)	NA
Protein coding genes	39	84 (76 unique)
tRNA	20	38 (20 unique)
rRNA	4 (3 unique)	8 (4 unique)
Coding genes with introns	9	11
tRNA with introns	0	8
# of predicted editing sites (PREP/PREPACT)	470/505	49/53
Editing sites/gene (PREP/PREPACT)	12/13	0.6/0.7
% of genome repeat elements	2.47%	3.05%

A total of 39 protein coding genes were annotated in the genome, in addition to 20 annotated tRNA and 4 rRNA genes (Fig. 1a, Table 2). The vast majority of the *E. grandis* mitochondrial genome is non-coding, with ~ 13% comprising of protein coding regions, and ~ 6% of introns. The mitochondrial protein coding genes are all single copy genes, with no duplications present (Table 2). The *E. grandis* mitochondrial genome does not contain any sequences similar to the ribosomal protein subunit genes *rps11*, *rps8*, and *rpl6*, which have been lost in angiosperms [15, 16]. Short fragments of *rps2* (141 nt) and *rps19* (42 and 69 nt) were found, but no full-length copies of these genes were present. The gene content was similar to other sequenced land plant mitochondrial genomes [17], with no genes exclusively lost in *E. grandis*. Ten *E. grandis* mitochondrial genes contain introns, with three of these, *nad1, nad2,* and *nad5*, being *trans*-spliced (Fig. 1a, Table 2). There are 16 single copy tRNA genes in the *E. grandis* mitochondrial genome, with two copies each of *tRNA-Asn*, *tRNA-Met*, *tRNA-Tyr* and *tRNA-fMet* (Fig. 1a, Table 1, Table 2)*.* The mitochondrial genome of *E. grandis* contains four rRNA genes, with two copies of *5S rRNA* present (Fig. 1a, Tables 1, 2).

**Table 2:** *E. grandis* mitochondrial genome gene content

Complex I	nad1 (5*)	nad2 (5*)	nad3	nad4 (4)	nad4L
	nad5 (4*)	nad6	nad7 (5)	nad9	
Complex III	cob				
Complex IV	cox1	cox2 (2)	cox3		
Complex V	atp1	atp4	atp6	atp8	atp9
Cytochrome C biogenesis	ccmB	ccmC	ccmFC (2)	ccmFN	
Ribosomal large subunit	rpl2 (2)	rpl5	rpl10	rpl16^a^	
Ribosomal small subunit	rps3 (2)	rps4	rps7	rps10 (2)	rps11
	rps12	rps13	rps14		
Intron maturase	matR				
Protein translocase	mttB				
Other	psbM^pl^				
rRNA genes	26S rRNA	18S rRNA	5S rRNA (×2)		
tRNA genes	tRNA-Asn (×2)^pl^	tRNA-Asp^pl^	tRNA-Cys	tRNA-fMet (×2)	tRNA-Gln
	tRNA-Glu^pl^	tRNA-Gly	tRNA-His^pl^	tRNA-Ile	tRNA-Lys
	tRNA-Met (×2)^pl^	tRNA-Phe	tRNA-Pro	tRNA-Ser	tRNA-Trp^pl^
	tRNA-Tyr (×2)^pl^				

Genes with multiple exons are denoted with the number of exons shown in parenthesis, and trans-spliced genes are indicated with *. tRNAs underlying plastid transferred regions are indicated with ^pl^. ^a^ Note that *rpl16* is annotated as a pseudogene due to an internal stop codon, but this gene has an in-frame GUG present downstream from the ATG start codon, which may be used as a start codon as in other plant species

Recently, the first mitochondrial genome of the order Myrtales was released, that of *Lagerstroemia indica* (NC_035616.1). Compared to the *E. grandis* mitochondrial genome, the 333,948 bp long mitochondrial genome of *L. indica* is smaller, with a higher GC content at 46% compared to 44% in *E. grandis*. Of the annotated *L. indica* mitochondrial genes, one has been lost in *E. grandis* (*rps19*), while two are not present in *L. indica* (*sdh3* and *rps13*). As is typical of land plant mitochondrial genomes [18], there has been massive re-arrangement of gene order between the two Myrtales families, with the largest block of collinear genes being *sdh4-cox3-atp8* (Additional file 5: Figure S2). Further, *rpl16* has gained an intron in *L. indica*, which is not present in *E. grandis*. Given the diverse nature of the Myrtales [19], and the frequent rearrangements and gene losses present in mitochondrial genomes [20] (Additional file 6: Figure S3), the differences between the two families are expected, and can be used in further phylogenetic analyses.

### Genome structure and gene content of the *E. grandis* plastid genome

**Fig. 2:**
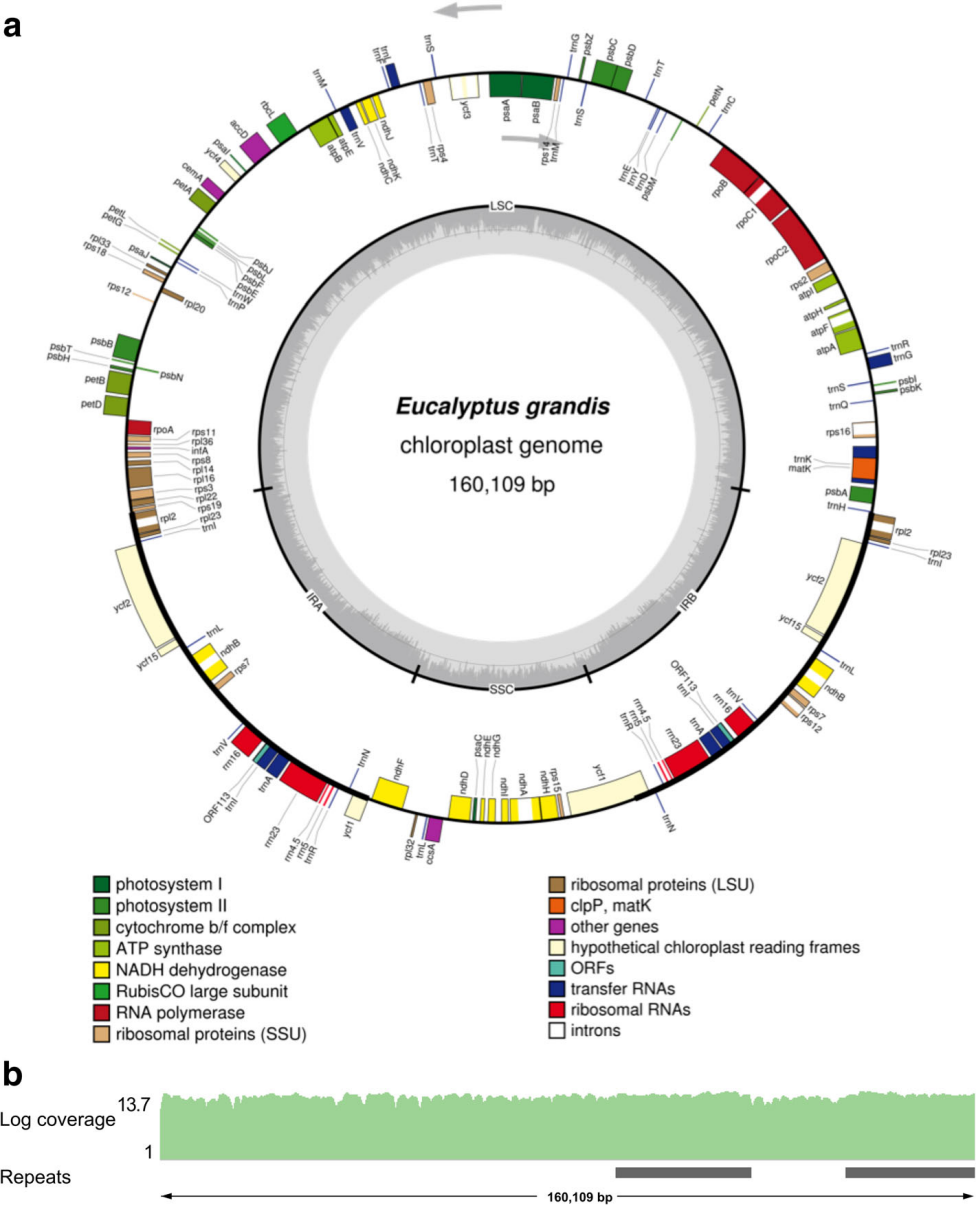
Plastid genome of *E. grandis*. **a**. Genomic features are shown facing outward (positive strand) and inward (negative strand) of the circular *E. grandis* plastid genome. The colour key shows the functional class of the plastid genes, and introns are shown in white. The GC content is represented in the innermost circle with the inverted repeat (IR) and single copy (SC) regions indicated. The figure was generated in OGDraw [77]. **b**. Genome coverage of *E. grandis* WGS reads in log2 scale (Log coverage) across the plastid genome. WGS reads were mapped with Bowtie 2 [78] and visualized in IGV [79]. The position of the plastid inverted repeat regions are shown below (Repeats) in grey

Although the plastid genome of *E. grandis* has been previously reported [21], some discrepancies in gene content exist when compared to other published *Eucalyptus* plastid genomes [22]. *Eucalyptus* plastid genomes typically contain 85 protein coding genes [22], and the available *E. grandis* plastid genome (NC_014570.1) contains 74 annotated protein coding genes [21, 23]. In the assembly reported here, the plastid genome of *E. grandis* was assembled using whole genome sequencing data (as above for the mitochondrial genome) and was subsequently annotated (Fig. 2a). The assembled plastid genome of *E. grandis* is 160,109 bp long, having the quadripartite structure of most land plant plastid genomes, with two large inverted repeat (IR) regions that are flanked by two single copy (SC) regions (small- SSC and large- LSC) (Fig. 2a). Coverage of the WGS reads aligned to the assembled plastid genome shows high coverage of 3500x across the length of the genome (Fig. 2b, Table 1). The high coverage of reads mapped give confidence in the downstream annotation and analysis of the assembled plastid genome.

The *E. grandis* plastid genome consists of 3.05% short repeat elements, the most abundant being simple and low complexity repeats (Table 1, Additional file 1: Table S1) The genome contains 90 genes, which includes six pseudogenes, for a total of 84 protein coding genes (Fig. 2a, Table 1). There are 37 annotated tRNA genes, representing 20 unique tRNAs. Introns are present in 8 of the annotated tRNA genes, namely *tRNA-Lys, tRNA-Gly, tRNA-Leu, tRNA-Val, tRNA-Ile* (2 copies), and *tRNA-Ala* (2 copies). The 8 rRNA genes in the plastid genome are found in the repeat regions, for a total of 4 unique rRNA genes. The intron structure of the plastid protein coding genes is highly conserved, with 11 genes containing at least two introns, of these, *ndhB* and *rpl12* are present as duplicates in the IR region. Three of the intron containing genes contain three exons; *ycf3*, *clpP*, and *rps12*. Two exons of *rps12* are present in the IR regions, and are trans-spliced to exon 1 found in the LSC region, as is common in land plants [24, 25]. The only difference in the coding regions of the previously published *Eucalyptus* plastid genomes (excluding the 2011 *E. grandis* plastid genome) is the annotation of *psbL*, which is annotated as a pseudogene, but has a predicted C to U editing site that creates a start codon (Additional file 7 - Sheet 2). The creation of a canonical start codon via C to U editing in the *psbL* gene has been well documented in other land plants [26, 27]. Thus, we include *psbL* as a bona fide gene in *E. grandis* plastid genome annotation.

### Post-transcriptional editing in the organellar genomes of *E. grandis*

Land plant plastid and mitochondrial encoded transcripts are known to undergo extensive post-transcriptional C to U editing, which generally results in non-synonymous amino acid changes, and can create and abolish start and stop codons [28]. In order to identify potential transcript editing sites in the *E. grandis* plastid and mitochondrial genomes, we predicted editing events using two homology based predictive approaches, PREPACT and PREP-suite [29, 30]. In the *E. grandis* mitochondrial genome, we identified 505 and 470 predicted C to U editing sites for an average of ~ 13 and ~ 12 editing sites per gene with PREPACT and PREP-mt respectively (Table 1, Additional file 8: Figure S4a, Additional file 7 - Sheet 1). Three of the predicted edits create canonical AUG translational start sites in mitochondrial *nad1A*, *nad4L*, and *rps10*, which have been reported in other plant species [31–33]. Interestingly, mitochondrial *rpl16* is annotated as a pseudogene due to an internal stop codon (TAG). In other plant species, this codon position is encoded as CAG and is post-transcriptionally edited to a stop codon (TAG), leading to a downstream non-canonical start codon (GTG) being used instead [32, 34]. This GTG is conserved in the mitochondrial genome of *E. grandis*, and it may be possible that *rpl16* is not a pseudogene and is translated from the GTG codon.

Plastid protein coding gene transcripts are also post-transcriptionally edited by C to U, although the frequency of editing sites in plastid genomes are drastically lower in land plant plastids compared to mitochondria [35]. In the plastid genome of *E. grandis*, we report 49 predicted C to U editing sites as predicted by PREPACT, using *Arabidopsis thaliana* as reference protein databases, and 53 using PREP-cp (Table 1, Additional file 8: Figure S4b, Additional file 7 - Sheet 2) [29]. These editing sites exclude sites duplicated in the inverted repeat regions, keeping only the sites found in IRA, as it includes the full length of *ycf1*. These results are standard for the highly conserved plastid genomes of land plants [36, 37].

We found evidence of editing sites in the organellar genomes of *E. grandis* with 24 bulked polyA-selected, paired end transcriptome datasets from eight *E. grandis* tissues (Additional file 8: Figure S4a and b). Using REDItools to discriminate between potential variants at the DNA level and true RNA editing sites, we could confirm 377 of the predicted mitochondrial editing sites, and 32 of the predicted plastid editing sites (Additional file 8: Figure S4 c and d, Additional file 7) [38]. These include the predicted start codons of *psbL*, *nad4L*, and *rps10*. REDItools identified 52 mitochondrial and 6 plastid edits not predicted by either PREPACT or PREP-suite, (Additional file 8: Figure S4c and d), which may be bona fide editing sites, or may be due the relatively low cut-offs defined in the analysis (total coverage > 10 reads, at least 3 reads supporting the edit). Further, REDItools identified synonymous editing sites in codon position 1 of plastid and mitochondrial genes, 1 of which is found in the plastid genome, and 6 in the mitochondrial genome (Additional file 7). Due to the fact that the transcriptome data was prepared from polyA selected RNA, the editing sites identified should be confirmed using total RNA sequencing, as polyadenylated transcripts in organelles are destined for degradation, and do not accurately reflect organellar transcriptomes [39, 40].

### DNA transfer between organellar and nuclear genomes

**Fig. 3:**
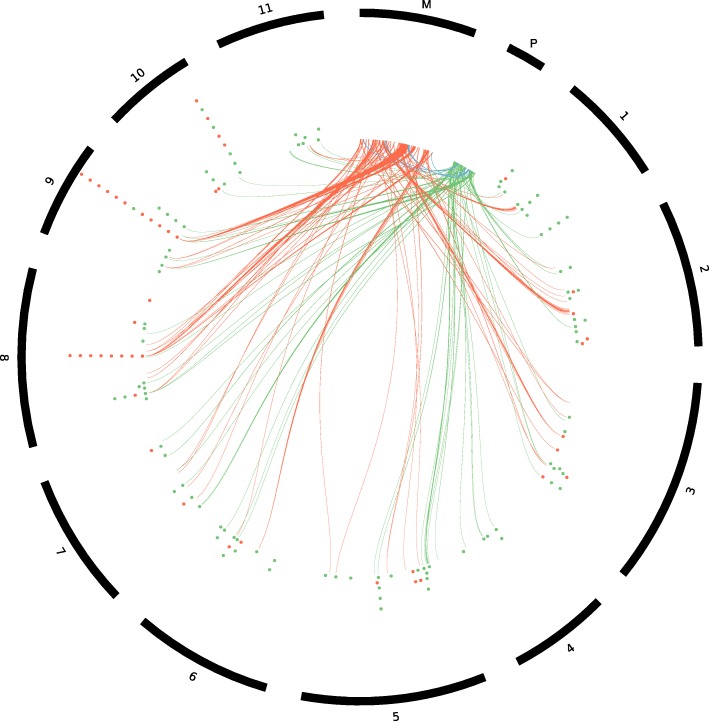
DNA and gene transfer between nuclear and organellar genomes in *E. grandis.* The outer track shows the relevant chromosomes of *E. grandis*, the inner track shows complete coding regions of NUMTs and NUPTs in red and green respectively. The red (mitochondria) and green (plastid) dots indicate full length gene transfers from the organelles to the nuclear genome. The ribbons represent DNA transfers identified by BLAST analysis greater than 500 nt, with percentage identity greater than 75%. Red ribbons indicate mitochondrial to nuclear DNA transfer, green ribbons indicate plastid to nuclear DNA transfer, and blue ribbons represent plastid to mitochondrial DNA transfer. For clarity, the scale of the plastid and mitochondrial genome size has been increased by 100x

In order to identify transferred DNA between the nuclear and organellar genomes of *E. grandis*, we used BLAST analysis to identify sequences of significant homology between the three genomes. After filtering the BLAST analysis results for sequences longer than 100 bp with e-values < 1 × 10^− 3^ and identity > 75%, we found a total of 751,886 bp of plastid origin and 1,256,558 bp of mitochondrial origin the nuclear genome (Fig. 3, Additional file 9: Table S2). The nuclear regions of organellar homology are distributed across all chromosomes of the nuclear genome (Fig. 3, Additional file 9: Table S2), with the largest proportion found on chromosome 5 for plastid DNA (88,691 bp), and chromosome 8 for mitochondrial DNA (193,727 bp). The mitochondrial genome of *E. grandis* consists of 6% (28,123 bp) chloroplast-like DNA sequences over 18 regions, with transfers ranging from 7281 bp to 152 bp in length. A single plastid gene, *psbM*, has been transferred and annotated in the *E. grandis* mitochondrial genome. We find that eight tRNA genes in the mitochondrial genome overlap with plastid transferred regions (indicated by ^pl^ in Table 2). BLAST analyses of the inter-organellar DNA transfers against all NCBI land plant organellar genomes show that inter-organellar DNA transfers are from the plastid to the mitochondria, and that no mitochondrial to plastid DNA transfer has taken place in *E. grandis* (Additional file 10: Table S3, Additional file 11).

Transferred DNA between the nuclear and organellar genomes of land plants creates the potential for complete transcript transfer that could be expressed from the nuclear genome [4]. In order to identify full length organellar transcripts in the nuclear genome of *E. grandis*, we used BLAST to align predicted organellar genes to *E. grandis* nuclear genes (> 80% of nuclear or organellar transcript length), and the annotation of the *E. grandis* v2.0 genome (Fig. 3, Additional file 12). We find 101 nuclear genes that have been transferred from the plastid genome (32 annotated as *A. thaliana* chloroplast genes, and 69 from the BLAST analysis). Further, there are 40 nuclear genes of mitochondrial origin (1 annotated as *A. thaliana* mitochondrial gene and 39 from the BLAST analysis). When genes without annotations in this group are examined for potential homologs in other plant species using the PLAZA database [41], we find that most of these nuclear genes are in fact orphan genes, with no homologs in the nuclear or organellar genomes of other dicot plant species. There are two exceptions, *Eucgr.J01097* and *Eucgr.J02736*, which are members of conserved gene families in plants. The first of these, *Eucgr.J01097,* is a homolog of a mitovirus RNA dependant polymerase [42], which occurs in the nuclear and mitochondrial genomes of 12 other dicot plant species (PLAZA family HOM03D004415) [34]. *Eucgr.J02736* forms part of a gene family that is present in six dicot plant species (PLAZA family HOM03006657) [41]. This gene is likely of plastid origin, as it is also found in the plastid transferred gene set (Additional file 12). Mismatches and indels present in the nuclear copies of full length organellar genes will allow for the identification of mRNA-seq reads mapped to the genome that they are expressed from (Additional file 12).

### Transcription of NUMT and NUPT genes in *E. grandis*

**Fig. 4:**
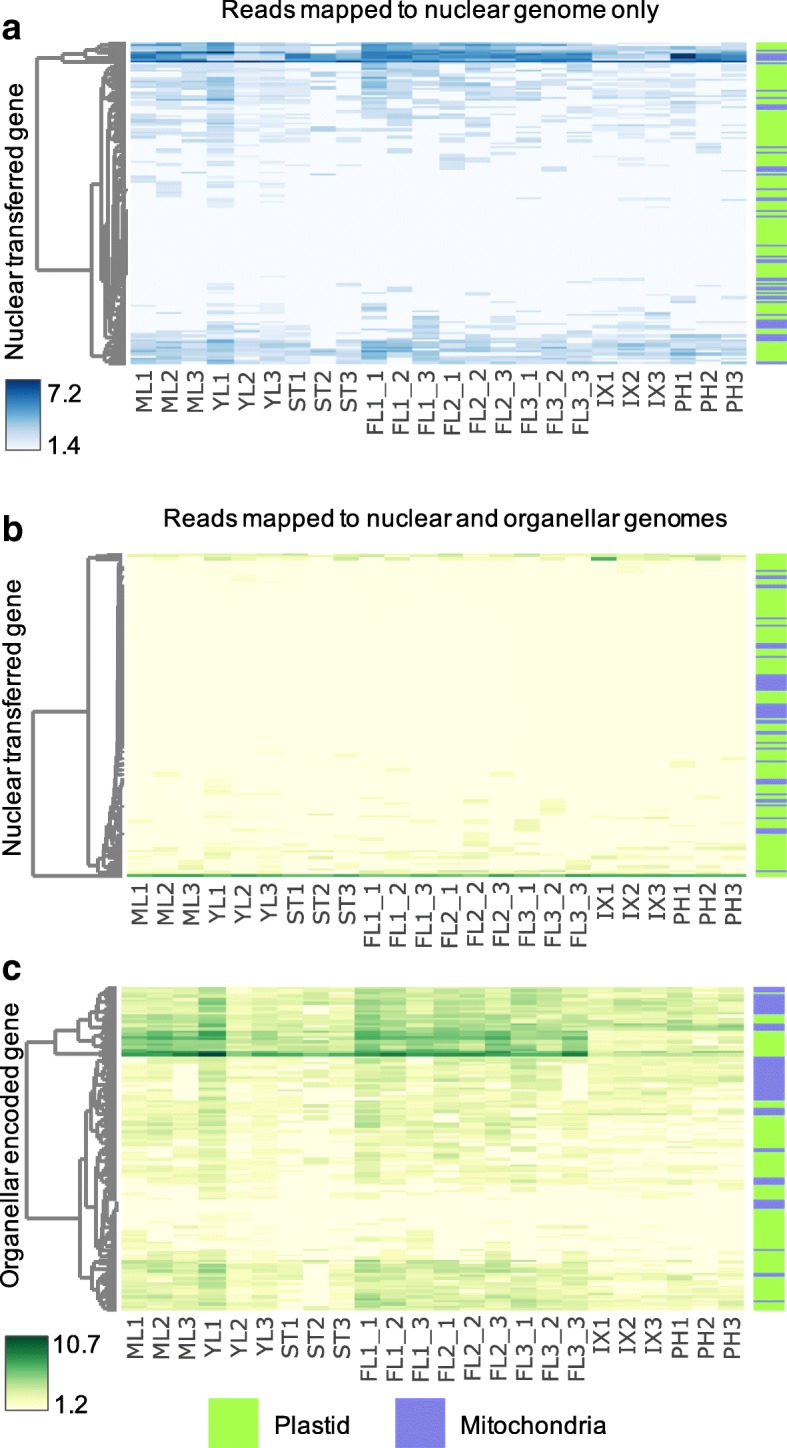
Poly-A selected RNA read abundance of nuclear genes with homology or annotation suggesting organellar transfer (**a**. and **b**.), and organellar encoded genes (**c**.) aligned to the nuclear genome only (blue) and the nuclear and organellar genomes of *E. grandis* (green). **a**. Variance stabilizing transformation (VST) counts of 141 organellar transferred genes in the nuclear genome of polyA selected RNA sequencing data aligned to the nuclear genome of *E. grandis* only. **b**. VST counts of full-length transferred genes in the nuclear genome of polyA selected RNA sequencing data aligned to the nuclear and organellar genomes of *E. grandis* simultaneously*.* **c**. VST counts of organellar encoded genes of polyA selected RNA sequencing data aligned to the nuclear and organellar genomes of *E. grandis* simultaneously. Row dendrograms on the left-hand side of all three heat maps show clustering of genes based on expression variation between tissues. Tissue samples are shown at the bottom edge of each heatmap, three biological replicates per tissue. Tissues are abbreviated as follows: Mature leaf (ML), young leaf (YL), shoot tips (ST), flowers stage 1 (FL_1), flowers stage 2 (FL_2), flowers stage 3 (FL_3), immature xylem (IX), and phloem (PH). The range of VST count values per heatmap are represented from low (white) to high (blue) for the polyA selected RNA mapping to the nuclear genome only, and from low (yellow) to high (green) for the polyA selected RNA mapping to the nuclear and organellar genomes. The bar on the right of the heatmaps shows the organellar origin of each gene, either plastid (transferred or encoded- green) or mitochondrial (transferred or encoded- blue)

In order to assess whether the *E. grandis* NUMTs and NUPTs identified above are functionally expressed, we aligned polyA-selected reads (from [43, 44]) to the nuclear genome, and compared read counts with the same reads aligned to the nuclear and organellar genomes (Fig. 4). To ensure that the reads aligned accurately to the organellar genomes, GSNAP was used with predicted organellar transcript editing sites defined as single nucleotide polymorphisms (SNPs) [45]. Evidence from eight tissues specific datasets revealed that organellar transferred genes in the nuclear genome are not functionally expressed (Fig. 4). The reads aligning to the nuclear genome (Fig. 4a) were drastically reduced when mapped to all three genomes simultaneously (Fig. 4b), and instead, mapped preferentially to the organellar genomes (Fig. 4c).

Of all the identified genes that are potentially transferred from the organellar genome to the nuclear genome, only one does not have decreased read counts when the polyA mRNA data is aligned to all three *E. grandis* genomes. This gene, *Eucgr.E01203*, was identified as a transferred gene due to its annotation as an *A. thaliana* chloroplast NADH-Ubiquinone/plastoquinone (complex I) protein gene (*ndhB2*). The parameters used in the BLAST analysis above did not identify this gene as an organellar transferred gene, as the CDS of this gene is truncated compared to the organellar *ndhB2* gene (*Eucgr.P00068*), with a length of 228 versus 1533 nt. Read coverage across this gene in mature leaf tissue shows that the aligned reads do not span the annotated CDS, rather, they are found in the 5’ UTR (Additional file 13: Figure S5). The variance stabilizing transformation (VST) counts of *Eucgr.E01203* are thus unlikely to represent functional gene expression.

Organellar encoded genes show that the polyA-selected mRNA reads aligned differentially across tissues in *E. grandis*. In general, the plastid and mitochondrial genes have low numbers of reads aligning across all tissues, with some genes having high numbers of reads aligning in leaf and flower tissues (Fig. 4c). Compared to the all nuclear encoded genes, we identified 28 organellar genes with significant polyA-selected read abundance variation between immature xylem and mature leaf tissues (Additional file 14: Table S4). All 28 of these organellar encoded genes have decreased polyadenylated transcripts in immature xylem as compared to mature leaf. Of these, only one is a mitochondrial encoded gene, *Eucgr.M00039* (*maturase R*). The plastid differentially polyadenylated genes are predominantly photosystem genes (*psaA, B,* and *J,* and *psbA, B, C, D, E, H, I, J, K, L,* and *T*). The tissue specific nature of the read abundance variation in the photosystem genes specifically shows that these reads are not an artefact of transcripts “escaping” polyA selection based their GC content [46]. Further, we conclude that plastid encoded photosystem genes are differentially polyadenylated between tissues in *E. grandis,* and that organellar encoded genes are either not significantly polyadenylated or are lowly expressed in xylem.

## Discussion

Organellar genomes are an important resource for many genomic and biotechnological applications [47], and as such, we aimed to provide a resource of high-quality sequences and annotations for the mitochondrial and plastid genomes of *Eucalyptus grandis*. The genus *Eucalyptus* consists of more than 700 species and their hybrids, many of which are economically and ecologically important [48, 49]. Additionally, *E. grandis* is an emerging model species for the study of xylogenesis [50]. The mitochondrial genome of *E. grandis* the second for the order Myrtales and should facilitate further studies in the phylogeny of this order [51, 52]. The size of the *E. grandis* mitochondrial genome, GC content, number of coding genes, and predicted RNA editing sites is well within the range of sequenced land plant mitochondrial genomes [53]. The mitochondrial genome of *E. grandis* shares many features with other published land plant mitochondrial genomes, specifically the loss of *rps* and *rpl* subunits [54]. The genome structure of the mitochondrial genome is potentially linear, or present as sub-genomic circles due to the presence of large repeat regions [20, 55]. We could identify one repeat mediated structural variant from the aligned paired-end reads, although any of the repeat regions could be involved in alternate conformations of the mitochondrial genome. As we could not confidently detect any other possible structural variants, mitochondrial DNA isolation from meristematic tissues or ovules [13, 55], and long-read sequencing methods may improve the assembly in future [56].

Organellar DNA is surprisingly mobile, and DNA transfers between organellar and nuclear genomes, and between species occur frequently [4], predominantly from the plastid and mitochondria to the nucleus [10], and from the nucleus and plastid to the mitochondria [9]. In many commercially important biomass crop species, large amounts of organellar DNA has been transferred to the nuclear genome. In *Populus trichocarpa* and *Gossypium raimondii*, near complete chloroplast and mitochondrial genomes respectively have been transferred to the nuclear genome [15, 57]. In *E. grandis*, we identified DNA transfers from the organelles to the nucleus, and from the plastid to the mitochondria (Fig. 3, Additional file 1: Table S1). Nuclear genes that align to the organellar genomes are gene fragments that have been annotated as complete genes due to the evidence of gene expression resulting from polyadenylated organellar transcripts. Using next-generation RNA-sequencing, we were able to show that the NUMT and NUPT genes, and nuclear genes which align to the organellar genomes are not functionally expressed from the nuclear genome of *E. grandis* (Fig. 4). Utilizing a method of SNP aware alignment, using predicted editing sites as SNPs, we show that reads in transferred regions preferentially align to the organellar genomes (Fig. 4). Further analysis showed that feature counts, especially when they are extremely low, do not accurately reflect transcript expression, but rather fragmented alignment of a few reads across the transcript (Additional file 13: Figure S5). This analysis allows for the confident alignment of mRNA reads to the three genomes of *E. grandis* for the quantification of organellar transcripts in future experiments.

The analysis of polyA-selected mRNA sequencing read alignment to the organellar genomes has value beyond identifying expressed NUMT and NUPT genes, as organellar genes are polyadenylated as a degradation signal [40, 58, 59]. We find that between mature leaf and immature xylem, the vast majority of differentially polyadenylated genes are photosystem genes from the chloroplast genome (Additional file 14: Table S4). Photosystem genes are either not expressed, or very lowly expressed in non-photosynthetic tissues such as xylem [60, 61]. Given RNA turnover requirements, and imprecise transcriptional termination, the highly expressed photosystem genes in chloroplasts may lead to the polyadenylation of those transcripts in mature leaf [40]. Additionally, mature leaf chloroplast transcriptomes are differentially regulated compared to those in young leaf [62], and transcript degradation may play a role in this process.

## Conclusion

This work provides a platform for further investigation into the myrtaceae by providing a reference genome and annotations for the mitochondria of *E. grandis*. The organellar genomes can be used in the future to study the transcription of organellar genes, and the tissue specific mechanism of transcriptional regulation by polyadenylation [5, 7]. Further, the co-evolution of nuclear and organellar genomes have been shown to affect hybrid vigour and speciation [63–66], and this work will allow for such studies in *Eucalyptus*, genera in which hybrids are ecologically and industrially important.

## Methods

### Assembly and annotation of the *E. grandis* organellar genomes

Paired end, whole genome sequencing reads of a three-year-old *E. grandis* genotype TAG0014 from mature leaf tissue was used in the assembly of the *E. grandis* mitochondrial and plastid genomes (SRP132546). The reads were sequenced by the Beijing Genomics Institute using the Illumina HiSeq 2000 platform. Contigs of mitochondrial origin, identified from the nuclear genome assembly project [12], were used as seed sequences for assembly using MITObim v1.6 using the -- quick flag, and kmer length of 41 [67]. The mitochondrial genome was assessed for circularity using the circules.py script available as a part of MITObim (https://github.com/chrishah/MITObim). SVDetect was used to determine if the WGS reads aligned to the mitochondrial genome assembly using Bowtie 2 showed evidence of alternative genome configurations [14, 68]. As they are mediated by large repeat regions, alternate configurations of the genome can be identified from discordant read pairs that mapped in the wrong orientation, or at a distance larger or smaller than half the insert size (< 250 bp, > 750 bp), which were identified using SAMtools v1.3.1 view flag ‘-F 1294’ [14, 69]. To avoid regions which may be artifacts of plastid and nuclear DNA transfer, we further removed all reads which were not uniquely mapped to the mitochondrial genome. The identified SVDetect breakpoints within 250 bp of a mitochondrial repeat region were identified using bedtools v2.27.1 closest [70].

The mitochondrial genome was manually annotated using a combination of homology-based predictions, namely Mitofy [71], MFannot [72], and Geneious v10.0.5 [73]. Similarly, the plastid genome was assembled using NOVOPlasty v1.1 with kmer length of 39 [74], with the previous *E. grandis* plastid genome as seed sequence (NC_014570.1). The plastid genome was manually annotated using DOGMA [75], CpGAVAS [76], Geneious v10.0.5 [73], and MFannot [72].

Transcript editing sites were identified using the PREPACT web server and PREP-suite (Mt and Cp) for both genomes [29, 30]. For PREPACT analysis of the mitochondrial genome, *Arabidopsis thaliana*, *Nicotiana tabacum*, and *Vitis vinifera* was used to identify conserved C to U edits using BLASTx prediction, with stop codons edited if possible and all other parameters kept at default. A predicted editing site was classified as being predicted by PREPACT if it occurred in at least two of the species used for prediction. For the PREPACT plastid genome editing site prediction, *Arabidopsis thaliana* was used as reference protein database for BLASTx prediction, with all other parameters kept at default. For PREP-suite analysis of the plastid and mitochondrial genomes, a prediction confidence cut-off of 0.5 was used to predict editing sites, with all other parameters at default. Low-complexity repeats were identified in both genomes using RepeatMasker [77], with reference set to *Arabidopsis thaliana*, and all other parameters as default. Large genomic repeats were identified with Unipro UGENE [78], with repeat identity set to > 95%, and repeat length > 100 nt. Both genomes were visualized with OrganellarGenomeDRAW [79], and WGS reads were aligned using Bowtie 2 [68] to visualize coverage using the Integrative Genomics Viewer (IGV [80]).

### Identification and analysis of NUMTs and NUPTs in the *E. grandis* nuclear genome

BLAST (BLAST 2.3.0+) hits of > 100 bp, e-value > 1 × 10^− 5^, and 75% identity were used in the analysis of NUPTs and NUMTs, and inter-organellar genome transfer [81]. Regions originating from the IR regions of the plastid genome were counted once, unless they spanned the SC flanking regions. Inter-organellar DNA transfers were assigned an organelle of origin using a custom BLAST database of all land plant organelles retrieved from GenBank in June 2017 [51]. Results of the DNA transfer analysis outlined above were visualized using Circos v0.69 [82] with transferred regions > 500 bp shown for clarity. In order to identify transferred protein coding genes between the nuclear and organellar genomes, BLAST analysis of full-length transcripts from the organellar genomes to the complete nuclear genome and vice versa was done. Transcripts are considered complete transfers if they covered > 80% of the transcript length in either the nuclear or organellar gene and had > 75% identity between transcripts. Nuclear genes that are annotated as organellar genes were identified based on their closest *A. thaliana* homolog from the *E. grandis* v2 nuclear genome annotation [12].

### PolyA-selected mRNA sequencing alignment, quantification, and editing analysis

PolyA-selected, paired end mRNA sequencing data from eight *E. grandis* tissues (as described in [43, 44]) were aligned to all three *E. grandis* genomes using GSNAP with allowed mismatch set to 1 (gmap v2016-09-23 [45]). Predicted editing sites of the organellar transcripts identified in the annotation step were used as SNP files for GSNAP alignment in order not to bias the alignment towards the nuclear genome. The resulting sam alignment files were converted to bam format using SAMtools view and sorted by position with SAMtools sort (SAMtools v1.3.1 [69]). The sorted bam files were then used to generate raw feature counts using HTSeq-count v0.6.1 [82] with concatenated nuclear and organellar gtf annotation files. DESeq2 v1.8.2 [83], implemented in RStudio v1.0.136 [84], was used to generated variance stabilized transformed (VST) counts and identify differentially expressed genes between immature xylem, phloem, and mature leaf tissue samples. The results were visualized using ggplot2 v2.2.1 [85] in RStudio v1.0.136.

REDItools version 1.0.4 [38] was used to identify editing sites using the aligned polyA selected reads across 8 *E. grandis* tissues. We used the REDItoolDnaRna.py script to ensure that organellar genomic variants were not called as editing sites due to transferred DNA regions, using the Bowtie 2 [68] genomic DNA alignments to differentiate between DNA variants and RNA editing [38]. The settings used were as follows: predict C to U and G to A edits (for sense and antisense genes, respectively), editing sites must have > 10 reads aligned, with > 3 reads supporting the editing event, minimum per base quality > 25. We then filtered the identified editing sites based on the following parameters: No DNA variants in the site, sense orientation with organellar gene coding regions (C to U for sense genes, and G to A for antisense genes). All tissue samples were bulked, and edits were identified if they were found in any dataset and were in codon position 1 or 2 of in the sense strand of plastid and mitochondrial genes.

## Additional files

Additional file 1: **Table S1.** *E. grandis* mitochondrial and plastid genome short repeat elements overview (DOCX 13 kb)

Additional file 2: Excel spreadsheet of the results of UniPro UGENE analysis of large (length > 100 bp, identity > 95%) repeats in the *Eucalyptus grandis* mitochondrial genome. (XLSX 10 kb)

Additional file 3: Excel spreadsheet of the results of SVDetect analysis results showing breakpoints of structural variants in the *E. grandis* mitochondrial genome (Sheet 1), and breakpoints within 250 bp of mitochondrial genome large repeat regions (Sheet 2). (XLSX 14 kb)

Additional file 4: **Figure S1**. Discordantly mapped read pairs flanking direct repeat 13 of the mitochondrial genome. The insert size of the reads is ~ 118,000 bp, compared to the expected 475. These reads suggest a repeat mediated structural variation, supported by SVDetect analysis. Read pair insert is shown by the red lines and the direct repeat is shown in the blue track. (PDF 51 kb)

Additional file 5: **Figure S2**. Mitochondrial genome gene order comparison between *Eucalyptus grandis* and *Lagerstroemia indica*. The gene order for the *E. grandis* mitochondrial genome is shown at the right of the figure, and that of *L. indica* on the top. Genes that are not found in each genome are indicated with red text. Collinear genes are indicated by red boxes. (PDF 29 kb)

Additional file 6: **Figure S3.** Multiple whole genome alignment of selected land plant mitochondrial genomes. Alignment was performed using the progressiveMauve algorithm in Mauve multiple alignment tool [87], with the coloured blocks representing Locally Collinear Blocks of sequences between genomes. The red lines indicate the length of the mitochondrial genomes, and the name of the organism is shown at the bottom of each genome. This figure shows the widespread genome rearrangements present in plant mitochondrial genomes. (PDF 286 kb)

Additional file 7: Excel spreadsheet of the results of predicted editing sites in the *E. grandis* mitochondrial (Sheet 1) and plastid (Sheet 2) genomes using PREPACT, PREP-suite, and REDItools, labelled by position of the edit in the coding sequence. (XLSX 38 kb)

Additional file 8: **Figure S4.** Number of predicted C to U editing sites in the mitochondrial and plastid genomes of *E. grandis* using PREPACT, PREP-suite, and REDITOOLS mRNA editing detection of polyA-selected reads. a. Number of editing sites (y-axis) in *E. grandis* mitochondrial genes (x-axis) as predicted by PREP-Mt (blue), PREPACT (orange), and evidence from bulked polyA-selected reads from three samples each of eight tissues in *E. grandis* using REDItools (DNA-RNA algorithm: minimum read depth = 10, minimum amount of reads per editing event = 3) shown in grey. b. Number of editing sites (y-axis) in *E. grandis* plastid genes (x-axis) as predicted by PREP-Cp (blue), PREPACT (orange), and evidence from polyA-selected reads using REDItools (grey). These figures show that bulked polyA selected reads are sufficient to detect the majority of predicted editing events in land plants, however the read depth lower than would be detected with total RNA sequencing. c. Number of predicted editing sites in common between PREP-Mt, PREPACT, and REDItools in the *E. grandis* mitochondrial genome. d. Number of predicted editing sites in common between PREP-Cp, PREPACT, and REDItools in the *E. grandis* plastid genome. (PDF 52 kb)

Additional file 9: **Table S2.** Amount of *E. grandis* organellar DNA transfer to nuclear chromosomes (DOCX 13 kb)

Additional file 10: **Table S3.** Inter-organellar DNA transfers in *E. grandis* show regions of high homology between *E. grandis* plastid and mitochondrial genomes. Additional BLAST analysis with land plant organellar genomes show that the transferred regions are all transferred from the plastid to the mitochondria (see Additional file 11). (DOCX 14 kb)

Additional file 11: Excel spreadsheet of the origin of inter-organellar DNA transfers, showing the organellar genomes of sequenced land plants, and the results of BLAST analysis of *E. grandis* mitochondrial genome (TAG0014_chr_M) regions that have significant homology to the *E. grandis* plastid genome. Note that all mitochondrial genomes analysed have no significant matches to the transferred regions, suggesting that there is no transfer of DNA from the mitochondrial genome to the plastid genome. (XLSX 667 kb)

Additional file 12: Excel spreadsheet of nuclear genes of organellar origin for mRNA-sequencing read mapping by homology using BLAST analysis (> 80% full length of gene matches with > 90% identity to organellar genome) or by annotation (annotation closest match is *Arabidopsis thaliana* organellar gene). (XLSX 13 kb)

Additional file 13: **Figure S5.** Sashimi plot of polyA-selected mRNA reads mapped to *Eucgr.E01203* in *E. grandis* mature leaf tissue. The plot shows the count of reads (0 to 105) aligned to the annotated gene regions of *Eucgr.E01203.* Reads were aligned using GSNAP and visualized in the Integrated Genome Viewer. Black lines show the annotated gene regions, and thicker black bars show the annotated protein coding regions. The plot shows that the read coverage of *Eucgr.E01203* across the protein coding regions is lower than in the 5’ UTR, indicating that the VST counts generated for this gene do not represent functional gene expression. (PNG 14 kb)

Additional file 14: **Table S4.** Differently expressed organellar encoded genes in *E. grandis* where negative log2 fold change values indicate increased polyA selected RNA read abundance in mature leaf compared to immature xylem. (DOCX 14 kb)

## Abbreviations

bp: Base pairs
IR: Inverted repeat
NUMT: Nuclear mitochondrial DNA
NUPT: Nuclear plastidial DNA
polyA: Polyadenylated
SC: Single copy
SNP: Single nucleotide polymorphism
UTR: Untranslated region
VST: Variance stabilizing transformation
WGS: Whole genome sequencing
