# Supplementary material for: The plastid and mitochondrial genomes of Eucalyptus grandis
Source: BMC Genomics. 2019 Feb 13;20:132. doi: 10.1186/s12864-019-5444-4 (PMC6373115; doi:10.1186/s12864-019-5444-4)
Supplement: Supplementary file 1 — Table S1. E. grandis mitochondrial and plastid genome short repeat elements overview (DOCX 13 kb) [file 12864_2019_5444_MOESM1_ESM.docx]

### Table S1

*E. grandis* mitochondrial and plastid genome short repeat elements overview

| Genome | Mitochondria | | Plastid | |
| --- | --- | --- | --- | --- |
| Total # of bases | 11 852 | | 4 880 | |
| % of genome | 2.47% | | 3.05% | |
| Repeat type | Number of elements | Total length | Number of elements | Total length |
| Ty1/Copia | 10 | 4 146 | 0 | 0 |
| Gypsy/DIRS1 | 5 | 1 967 | 2 | 72 |
| Small RNA | 11 | 2 881 | 6 | 2 388 |
| Simple repeats | 69 | 2 502 | 43 | 1 895 |
| Low complexity repeats | 9 | 356 | 12 | 525 |
