# Supplementary figures and images for: The plastid and mitochondrial genomes of Eucalyptus grandis
Source: BMC Genomics. 2019 Feb 13;20:132. doi: 10.1186/s12864-019-5444-4 (PMC6373115; doi:10.1186/s12864-019-5444-4)

Repeat units

Coverage histogram

Reads aligned discordantly  
to mitochondrial genome

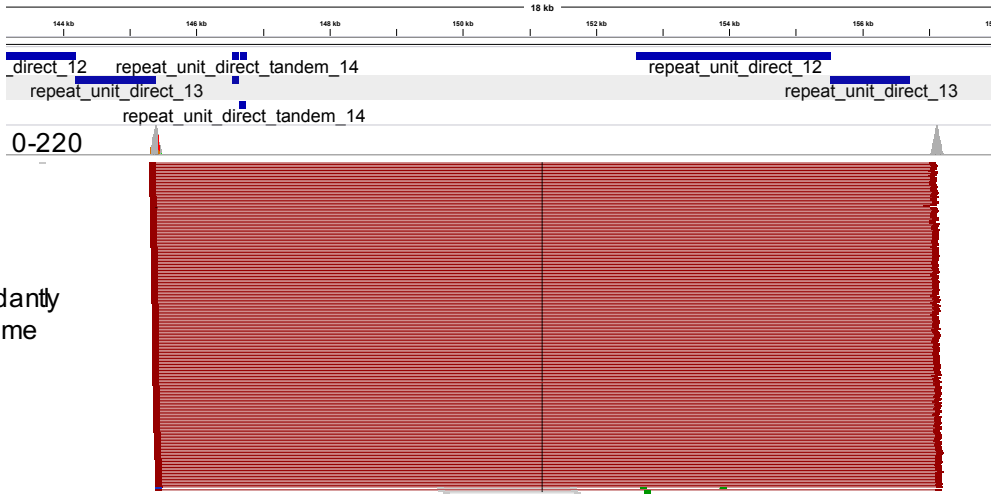

Supplement: Supplementary file 4 — Figure S1. Discordantly mapped read pairs flanking direct repeat 13 of the mitochondrial genome. The insert size of the reads is ~ 118,000 bp, compared to the expected 475. These reads suggest a repeat mediated structural variation, supported by SVDetect analysis. Read pair insert is shown by the red lines and the direct repeat is shown in the blue track. (PDF 51 kb) [file 12864_2019_5444_MOESM4_ESM.pdf]

# Lagerstroemia indica mitochondrial genes

*Eucalyptus grandis* mitochondrial genes

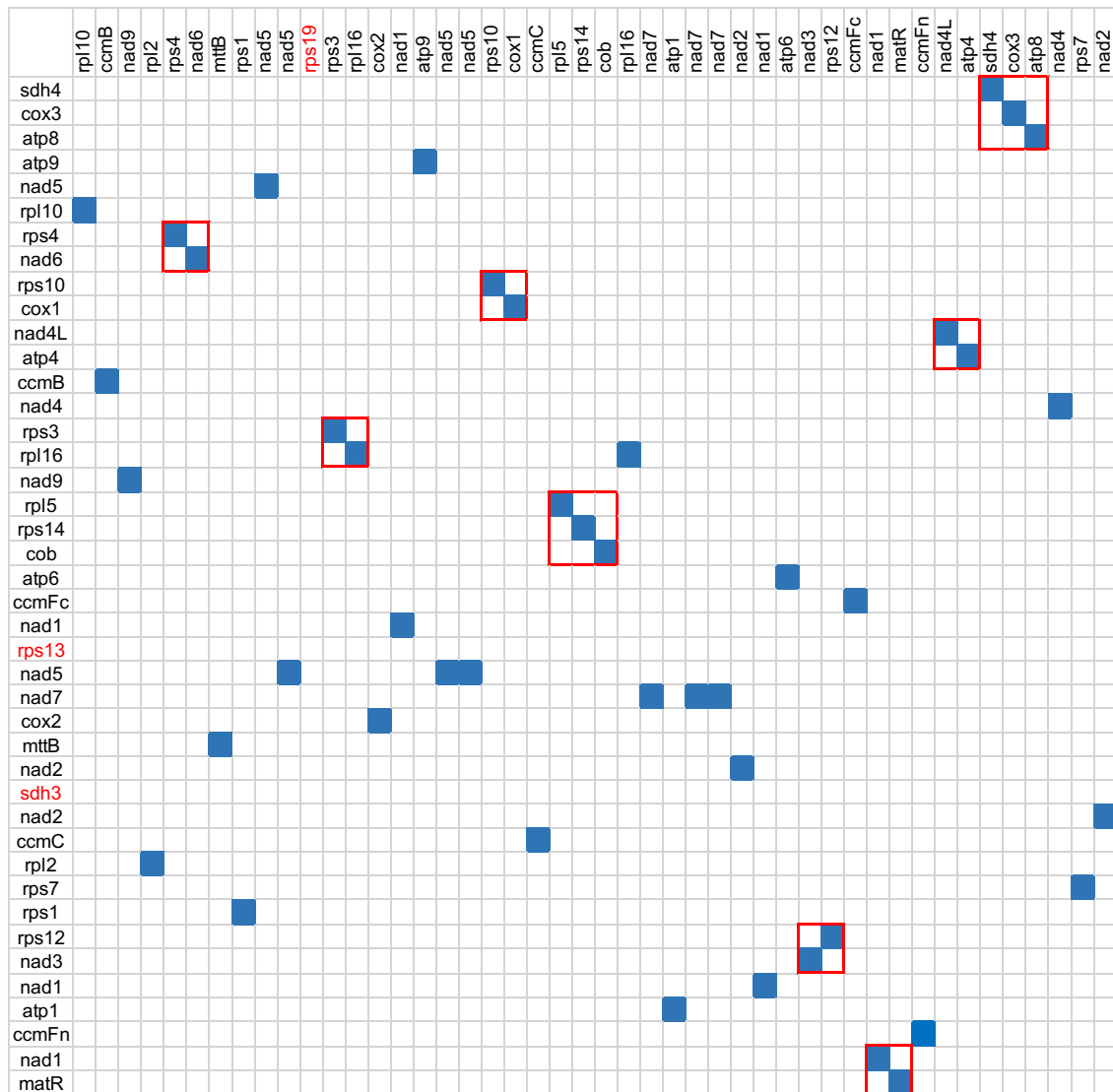

Supplement: Supplementary file 5 — Figure S2. Mitochondrial genome gene order comparison between Eucalyptus grandis and Lagerstroemia indica. The gene order for the E. grandis mitochondrial genome is shown at the right of the figure, and that of L. indica on the top. Genes that are not found in each genome are indicated with red text. Collinear genes are indicated by red boxes. (PDF 29 kb) [file 12864_2019_5444_MOESM5_ESM.pdf]

a.

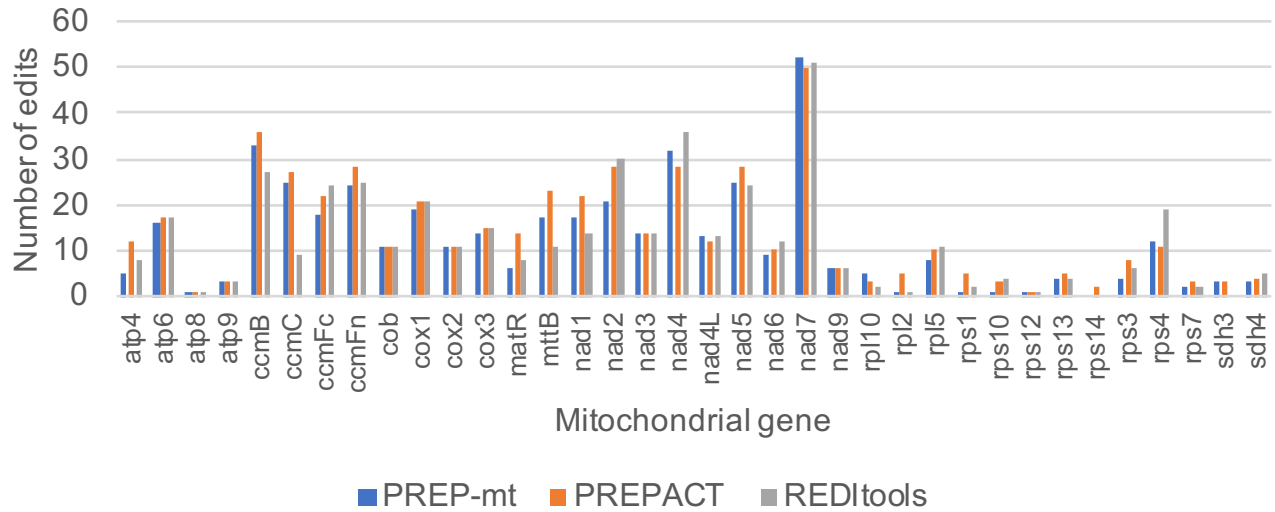

b.

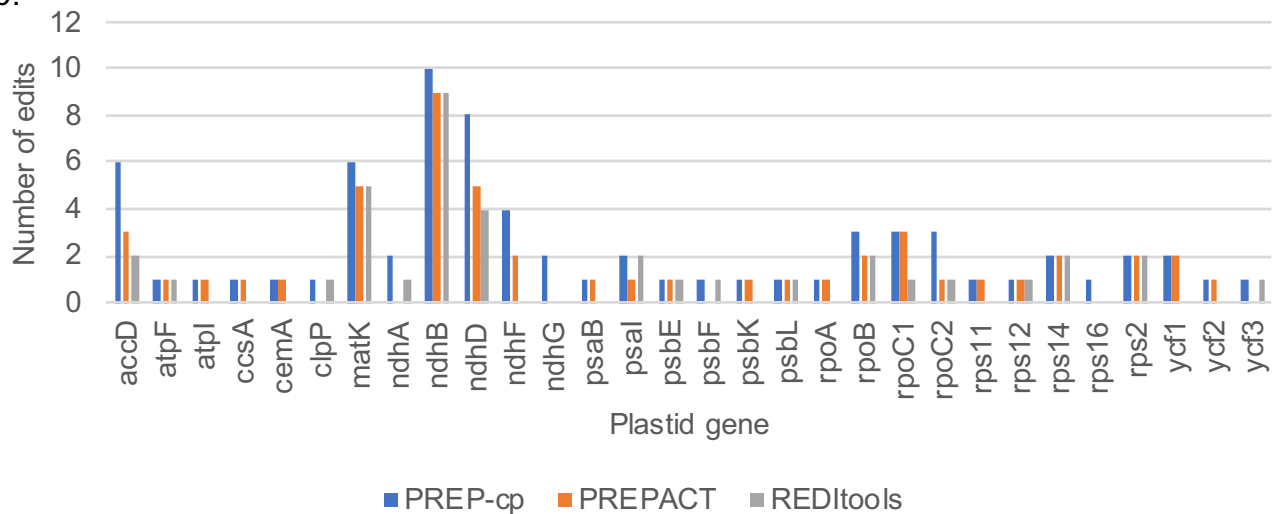

c.

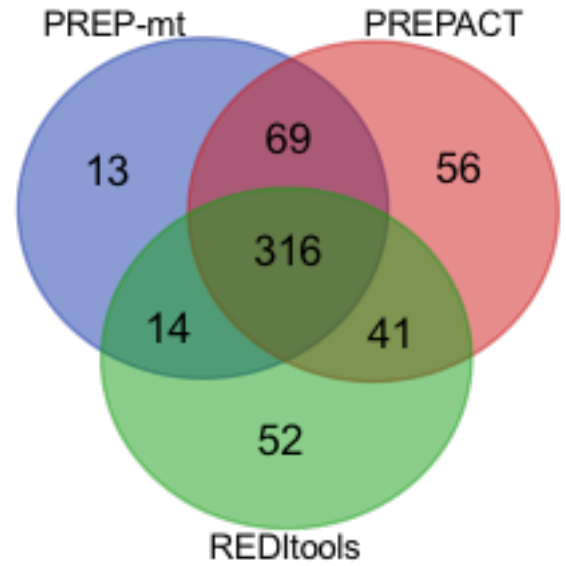

d.

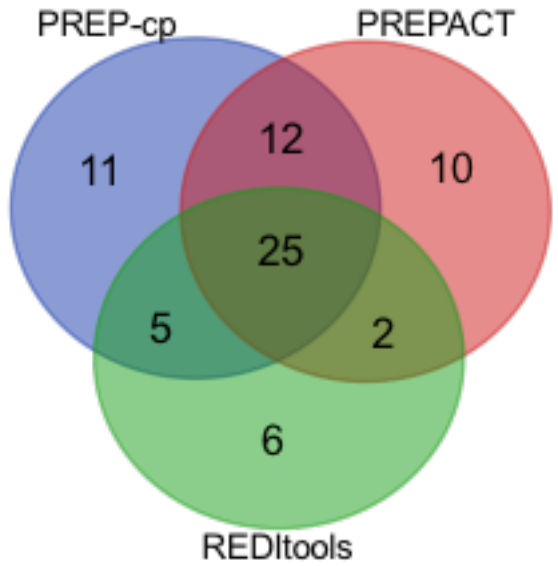

Supplement: Supplementary file 8 — Figure S4. Number of predicted C to U editing sites in the mitochondrial and plastid genomes of E. grandis using PREPACT, PREP-suite, and REDITOOLS mRNA editing detection of polyA-selected reads. a. Number of editing sites (y-axis) in E. grandis mitochondrial genes (x-axis) as predicted by PREP-Mt (blue), PREPACT (orange), and evidence from bulked polyA-selected reads from three samples each of eight tissues in E. grandis using REDItools (DNA-RNA algorithm: minimum read depth = 10, minimum amount of reads per editing event = 3) shown in grey. b. Number of editing sites (y-axis) in E. grandis plastid genes (x-axis) as predicted by PREP-Cp (blue), PREPACT (orange), and evidence from polyA-selected reads using REDItools (grey). These figures show that bulked polyA selected reads are sufficient to detect the majority of predicted editing events in land plants, however the read depth lower than would be detected with total RNA sequencing. c. Number of predicted editing sites in common between PREP-Mt, PREPACT, and REDItools in the E. grandis mitochondrial genome. d. Number of predicted editing sites in common between PREP-Cp, PREPACT, and REDItools in the E. grandis plastid genome. (PDF 52 kb) [file 12864_2019_5444_MOESM8_ESM.pdf]

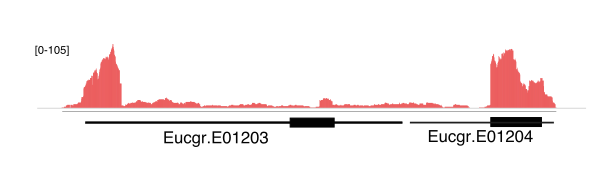

Supplement: Supplementary file 13 — Figure S5. Sashimi plot of polyA-selected mRNA reads mapped to Eucgr.E01203 in E. grandis mature leaf tissue. The plot shows the count of reads (0 to 105) aligned to the annotated gene regions of Eucgr.E01203. Reads were aligned using GSNAP and visualized in the Integrated Genome Viewer. Black lines show the annotated gene regions, and thicker black bars show the annotated protein coding regions. The plot shows that the read coverage of Eucgr.E01203 across the protein coding regions is lower than in the 5’ UTR, indicating that the VST counts generated for this gene do not represent functional gene expression. (PNG 14 kb) [file 12864_2019_5444_MOESM13_ESM.png]
