# Supplementary material for: The plastid and mitochondrial genomes of Eucalyptus grandis
Source: BMC Genomics. 2019 Feb 13;20:132. doi: 10.1186/s12864-019-5444-4 (PMC6373115; doi:10.1186/s12864-019-5444-4)
Supplement: Supplementary file 6 — Figure S3. Multiple whole genome alignment of selected land plant mitochondrial genomes. Alignment was performed using the progressiveMauve algorithm in Mauve multiple alignment tool [87], with the coloured blocks representing Locally Collinear Blocks of sequences between genomes. The red lines indicate the length of the mitochondrial genomes, and the name of the organism is shown at the bottom of each genome. This figure shows the widespread genome rearrangements present in plant mitochondrial genomes. (PDF 286 kb) [file 12864_2019_5444_MOESM6_ESM.pdf]

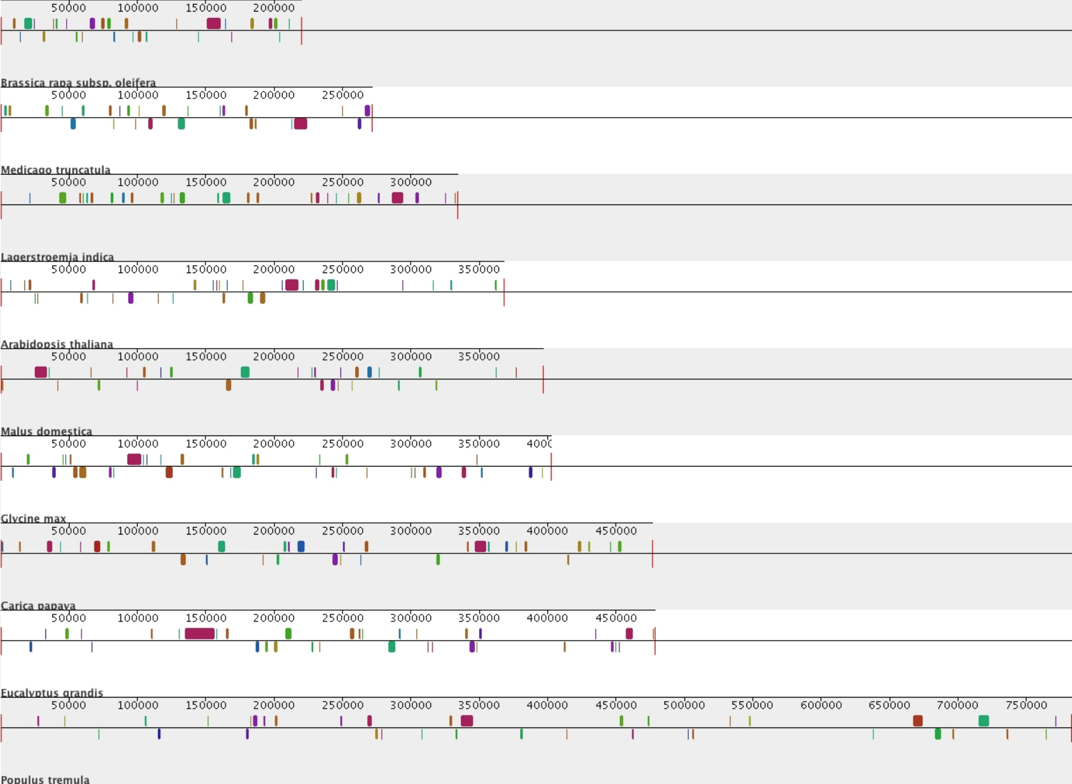

50000 100000 150000 200000
Brassica rapa subsp. oleifera
50000 100000 150000 200000 250000
Medicago truncatula
50000 100000 150000 200000 250000 300000
Lagerstroemia indica
50000 100000 150000 200000 250000 300000 350000
Arabidopsis thaliana
50000 100000 150000 200000 250000 300000 350000
Malus domestica
50000 100000 150000 200000 250000 300000 350000
Glycine max
50000 100000 150000 200000 250000 300000 350000 400000 450000
Carica papaya
50000 100000 150000 200000 250000 300000 350000 400000 450000
Eucalyptus grandis
50000 100000 150000 200000 250000 300000 350000 400000 450000 500000 550000 600000 650000 700000 750000
Populus tremula
