# Supplementary material for: The plastid and mitochondrial genomes of Eucalyptus grandis
Source: BMC Genomics. 2019 Feb 13;20:132. doi: 10.1186/s12864-019-5444-4 (PMC6373115; doi:10.1186/s12864-019-5444-4)
Supplement: Supplementary file 9 — Table S2. Amount of E. grandis organellar DNA transfer to nuclear chromosomes (DOCX 13 kb) [file 12864_2019_5444_MOESM9_ESM.docx]

### Table S2

Amount of *E. grandis* organellar DNA transfer to nuclear chromosomes

| Chromosome | Mitochondrial bp transferred | Plastid bp transferred |
| --- | --- | --- |
| Chr01 | 110 893 | 58 447 |
| Chr02 | 126 693 | 55 925 |
| Chr03 | 122 324 | 67 204 |
| Chr04 | 48 062 | 40 511 |
| Chr05 | 113 254 | 88 691 |
| Chr06 | 69 555 | 64 774 |
| Chr07 | 76 350 | 71 287 |
| Chr08 | 193 727 | 73 264 |
| Chr09 | 180 315 | 73 560 |
| Chr10 | 57 747 | 57 472 |
| Chr11 | 63 092 | 54 441 |
| Scaffolds | 94 546 | 53 742 |
| Total | 1 256 558 | 759 318 |
