# Supplementary material for: The plastid and mitochondrial genomes of Eucalyptus grandis
Source: BMC Genomics. 2019 Feb 13;20:132. doi: 10.1186/s12864-019-5444-4 (PMC6373115; doi:10.1186/s12864-019-5444-4)
Supplement: Supplementary file 10 — Table S3. Inter-organellar DNA transfers in E. grandis show regions of high homology between E. grandis plastid and mitochondrial genomes. Additional BLAST analysis with land plant organellar genomes show that the transferred regions are all transferred from the plastid to the mitochondria (see Additional file 11). (DOCX 14 kb) [file 12864_2019_5444_MOESM10_ESM.docx]

### Table S3

Inter-organellar DNA transfers in *E. grandis* show regions of high homology between *E. grandis* plastid and mitochondrial genomes. Additional BLAST analysis with land plant organellar genomes show that the regions are all transferred from the plastid to the mitochondria (see Additional file 4).

| Query | Subject | % Identity | Length | e-value | Origin |
| --- | --- | --- | --- | --- | --- |
| *E. grandis* mitochondria | *E. grandis* plastid | 99.99 | 7 282 | 0.00E+00 | Plastid |
| *E. grandis* mitochondria | *E. grandis* plastid | 98.51 | 4 616 | 0.00E+00 | Plastid |
| *E. grandis* mitochondria | *E. grandis* plastid | 99.46 | 3 924 | 0.00E+00 | Plastid |
| *E. grandis* mitochondria | *E. grandis* plastid | 98.7 | 1 851 | 0 | Plastid |
| *E. grandis* mitochondria | *E. grandis* plastid | 99.93 | 1 360 | 0 | Plastid |
| *E. grandis* mitochondria | *E. grandis* plastid | 99.04 | 1 350 | 0 | Plastid |
| *E. grandis* mitochondria | *E. grandis* plastid | 90.77 | 1 626 | 0 | Plastid |
| *E. grandis* mitochondria | *E. grandis* plastid | 100 | 1 035 | 0 | Plastid |
| *E. grandis* mitochondria | *E. grandis* plastid | 99.81 | 1 039 | 0 | Plastid |
| *E. grandis* mitochondria | *E. grandis* plastid | 100 | 768 | 0 | Plastid |
| *E. grandis* mitochondria | *E. grandis* plastid | 100 | 688 | 0 | Plastid |
| *E. grandis* mitochondria | *E. grandis* plastid | 100 | 653 | 0 | Plastid |
| *E. grandis* mitochondria | *E. grandis* plastid | 99.8 | 512 | 0 | Plastid |
| *E. grandis* mitochondria | *E. grandis* plastid | 97.79 | 272 | 8E-131 | Plastid |
| *E. grandis* mitochondria | *E. grandis* plastid | 97.41 | 232 | 1E-108 | Plastid |
| *E. grandis* mitochondria | *E. grandis* plastid | 98.7 | 154 | 2E-72 | Plastid |
| *E. grandis* mitochondria | *E. grandis* plastid | 78.76 | 452 | 2E-67 | Plastid |
| *E. grandis* mitochondria | *E. grandis* plastid | 75.06 | 429 | 5E-48 | Plastid |
