# Supplementary material for: The plastid and mitochondrial genomes of Eucalyptus grandis
Source: BMC Genomics. 2019 Feb 13;20:132. doi: 10.1186/s12864-019-5444-4 (PMC6373115; doi:10.1186/s12864-019-5444-4)
Supplement: Supplementary file 14 — Table S4. Differently expressed organellar encoded genes in E. grandis where negative log2 fold change values indicate increased polyA selected RNA read abundance in mature leaf compared to immature xylem. (DOCX 14 kb) [file 12864_2019_5444_MOESM14_ESM.docx]

### Table S4

Differently expressed organellar encoded genes in *E. grandis* where negative log2 fold change values indicate increased polyA selected RNA read abundance in mature leaf compared to immature xylem.

| Nuclear gene ID | log2 fold change | p-value | p-adjusted |
| --- | --- | --- | --- |
| Eucgr.P000048 | -5.68495009 | 2.03E-14 | 2.81E-13 |
| Eucgr.P000017 | -6.377806109 | 1.38E-13 | 1.76E-12 |
| Eucgr.P000021 | -5.253655908 | 5.60E-10 | 4.75E-09 |
| Eucgr.P000020 | -4.72825449 | 2.03E-09 | 1.60E-08 |
| Eucgr.P000004 | -4.929300965 | 2.48E-09 | 1.93E-08 |
| Eucgr.P000008 | -3.464742899 | 5.66E-08 | 3.64E-07 |
| Eucgr.P000006 | -4.464032842 | 1.58E-07 | 9.47E-07 |
| Eucgr.P000001 | -4.089175253 | 1.88E-07 | 1.11E-06 |
| Eucgr.P000007 | -4.014735664 | 2.05E-07 | 1.21E-06 |
| Eucgr.P000009 | -4.818293723 | 4.42E-06 | 2.12E-05 |
| Eucgr.P000019 | -4.337642569 | 1.15E-05 | 5.16E-05 |
| Eucgr.P000005 | -5.830750151 | 1.46E-05 | 6.43E-05 |
| Eucgr.P000051 | -5.305500857 | 1.63E-05 | 7.14E-05 |
| Eucgr.P000038 | -5.461236684 | 2.51E-05 | 1.06E-04 |
| Eucgr.P000029 | -3.129162319 | 4.96E-05 | 1.99E-04 |
| Eucgr.P000016 | -4.110545824 | 6.70E-05 | 2.62E-04 |
| Eucgr.P000030 | -2.781186404 | 9.89E-05 | 3.74E-04 |
| Eucgr.P000035 | -4.63894886 | 1.67E-04 | 6.06E-04 |
| Eucgr.M000039 | -2.611720867 | 6.91E-04 | 2.20E-03 |
| Eucgr.P000049 | -4.290770148 | 3.92E-03 | 1.03E-02 |
| Eucgr.P000056 | -3.993676505 | 4.58E-03 | 1.19E-02 |
| Eucgr.P000062 | -2.185109271 | 6.34E-03 | 1.58E-02 |
| Eucgr.P000041 | -3.967222852 | 7.12E-03 | 1.76E-02 |
| Eucgr.P000036 | -3.843149162 | 7.68E-03 | 1.88E-02 |
| Eucgr.P000055 | -2.26024327 | 8.10E-03 | 1.97E-02 |
| Eucgr.P000075 | -2.944863496 | 8.81E-03 | 2.11E-02 |
| Eucgr.P000034 | -3.678466272 | 1.13E-02 | 2.62E-02 |
| Eucgr.P000072 | -3.234384168 | 1.97E-02 | 4.28E-02 |
